# Case Report: A hidden cause of severe hemorrhage: a diagnostic and therapeutic challenge

**DOI:** 10.3389/fimmu.2026.1791266

**Published:** 2026-04-29

**Authors:** Ziying Han, Jing Ruan, Li Wang, Wei Wang, Tienan Zhu

**Affiliations:** 1 General Internal Medicine, Peking Union Medical College Hospital, Chinese Academy of Medical Sciences & Peking Union Medical College, Beijing, China; 2 Department of Hematology, Peking Union Medical College Hospital, Chinese Academy of Medical Sciences & Peking Union Medical College, Beijing, China; 3 Department of Rheumatology and Clinical Immunology, Key Laboratory of Rheumatology and Clinical Immunology, Ministry of Education, Peking Union Medical College Hospital, Peking Union Medical College, Chinese Academy of Medical Sciences, National Clinical Research Center for Dermatologic and Immunologic Diseases, Beijing, China; 4 Department of Rheumatology and Immunology, Peking University International Hospital, Beijing, China

**Keywords:** acquired factor XIII deficiency, coagulation disorders, hematology, hemorrhagic shock, systemic lupus erythematosus

## Abstract

Acquired factor XIII (FXIII) deficiency is a rare but life-threatening cause of hemorrhagic diseases. We report a case of a patient presenting with life-threatening bleeding with normal routine coagulation tests. The diagnosis was confirmed by a specific FXIII activity assay. The subsequent absence of ANA delayed the definitive diagnosis, which was ultimately established as ANA-negative systemic lupus erythematosus (SLE) based on comprehensive clinical criteria and multisystem involvement. This case presented a profound diagnostic dilemma, requiring not only the identification of a rare acquired FXIII deficiency as the cause of unexplained bleeding, but also the confirmation of SLE despite seronegativity, relying on its multisystem manifestations.

## Introduction

Acquired factor XIII (FXIII), a crucial enzyme in the coagulation cascade, plays a pivotal role in stabilizing fibrin clots during traumatic bleeding and wound healing. Deficiency of FXIII may arise from either excessive consumption or insufficient synthesis of this zymogen. Acquired FXIII deficiency is a rare disorder which predominantly occurs secondary to autoimmune disorders, hematological malignancies, major surgical procedures, traumatic injuries and so on. Systemic lupus erythematosus (SLE) is typically screened for with antinuclear antibody (ANA) testing, but ANA-negative SLE, while uncommon, is a well-recognized clinical variant. The coexistence of acquired FXIII deficiency and ANA-negative SLE has not been previously reported. Herein, we report a case of acquired FXIII deficiency presenting with severe bleeding, whose evaluation revealed multisystem manifestations leading to the diagnosis of ANA-negative SLE.

## Case presentation

A 54-year-old woman was admitted in the emergency with severe persistent left lower abdominal pain for two days. Laboratory analysis demonstrated normocytic anemia (hemoglobin 90 g/L, MCV 93.6 fL) accompanied by thrombocytopenia (68×10^9^/L). Coagulation studies revealed mild prolongation of prothrombin time (PT, 15.8 s) and activated partial thromboplastin time (APTT, 29.6 s). Impaired renal function was demonstrated: serum creatinine 120 μmol/L; urinalysis showed significant proteinuria (>3 g/L) and hematuria with 100% dysmorphic red blood cells. Key laboratory results were showed in **Table 1**.

**Table 1:** Key laboratory findings on admission.

Test Items	Results	Reference range	Detection method
WBC	4.25×10^9^/L	3.50-9.50×10^9^/L	Flow cytometry + nucleic acid fluorescent staining.
NEUT	2.44×10^9^/L	2.00-7.50×10^9^/L	
HGB	90 g/L	110-150g/L	Photoelectric colorimetry
PLT	68×10^9^/L	100-350×10^9^/L	Sheath flow impedance method
PT	15.8s	10.4-12.6s	Coagulation method
APTT	29.6s	23.3-32.5s	
D-dimer	0.55mg/L FEU	0-0.55 mg/L FEU	Immunoturbidimetry
C3	0.199g/L	0.730-1.460g/L	
C4	0.025g/L	0.100-0.400 g/L	
LA	0.92	<1.20	Coagulation method (dRVVT)
ANA	Negative	Negative	Indirect immunofluorescence
Antiphospholipid antibody*	Negative	Negative	Chemiluminescent assay
FXIII	Positive	Negative	Urea clot solubility test

*Antiphospholipid antibody test include anti-cardiolipin antibody IgA/IgM/IgG and anti-β2-glycoprotein 1 antibody IgA/IgM/IgG.

**Figure 1:**
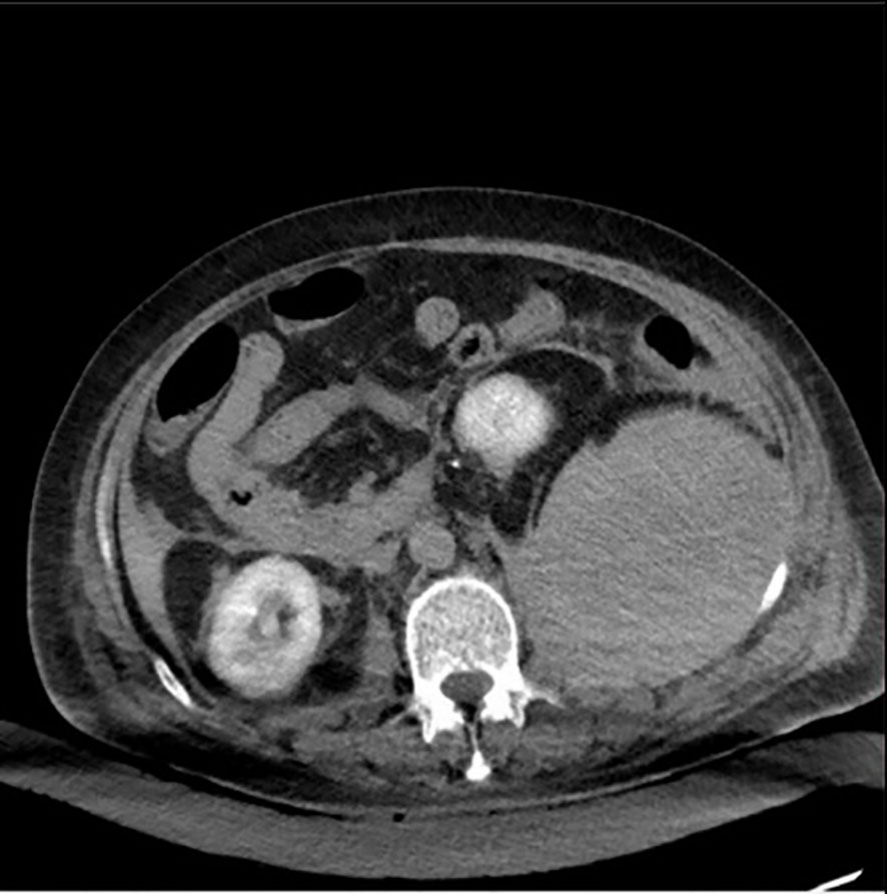
CT suggests a massive hematoma posterior to the left psoas major.

Contrast-enhanced abdominal CT identified pleural effusion which was subsequently confirmed as hemorrhagic exudate following drainage, accompanied by multiple hypodense lesions posterior to the left psoas major, with the largest measuring 11.2×10.1 cm, radiologically consistent with multifocal hemorrhage (**Figure 1**). Subsequent diagnostic celiac artery angiography failed to demonstrate active contrast extravasation. During hospitalization, the patient developed hemorrhagic shock secondary to progressive hemoglobin depletion, reaching a critical nadir of 38 g/L.

Since routine coagulation studies were only slightly abnormal, screening test for factor XIII (FXIII) was performed. The urea clot solubility test was positive in this patient; therefore, a correction study was subsequently performed. The patient’s citrated plasma clot demonstrated complete dissolution within 13 minutes, while the normal control plasma clot remained intact at 24-hour observation, indicating FXIII deficiency. We further did 1:1 mixing study with normal plasma which showed persistent clot dissolution in this patient, indicating the presence of a FXIII inhibitor. Due to technical limitations, further measurement of FXIII activity was not performed in this patient (1, 2). However, based on the patient’s clinical manifestations and the FXIII assay mentioned above, we believe that the diagnosis of acquired FXIII deficiency can be established.

**Figure 2:**
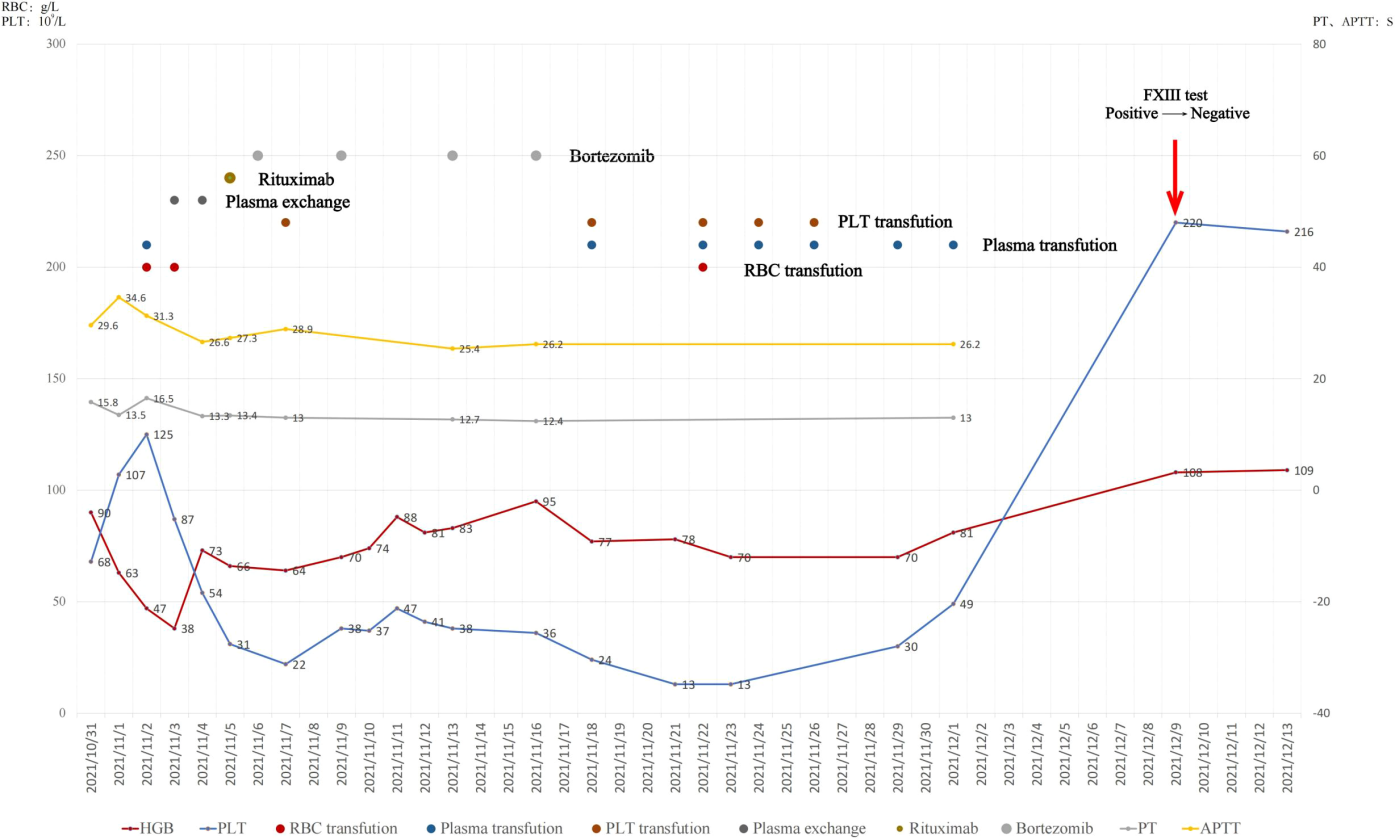
Dynamic changes in hemoglobin, platelet count, and coagulation parameters during hospitalization, with annotations of transfusions and therapeutic interventions. Conversion of the urea clot solubility test from positive to negative after treatment is indicated (red arrow).

The patient was transferred to the ICU and received carbazochrome sodium sulfonate for hemostasis along with transfusion of fresh frozen plasma FFP. Plasma exchange therapy was initiated immediately, even though the underlying cause had not yet been identified. Meanwhile, based on the experience of our hematology department, a regimen combining rituximab and bortezomib was also initiated. This protocol can rapidly clear antibodies and carries a lower risk of infection compared to the traditional first-line treatment of high-dose steroids plus cyclophosphamide (3). Key results and treatments were shown in **Figure 2**. However, repeat abdominal CT showed slight hematoma enlargement with a progressive decrease of platelet count to 13×10^9^/L.

However, the precise etiology of this patient’s FXIII deficiency remains undetermined. Upon reviewing the patient’s medical history, the patient reported a 7-month history of recurrent fever with rash and oral ulceration, which manifested 14 days following administration of the COVID-19 vaccine. Previous glucocorticoid therapy provided transient fever resolution, while antibiotic treatments proved ineffective. Three months prior to admission, she experienced recurrence of fever and developed diabetic ketoacidosis and Hashimoto’s thyroiditis, followed by alopecia, spontaneous gingival bleeding, oral mucosal hematomas, and widespread cutaneous ecchymoses.

The patient’s clinical manifestations and immunological profile suggested potential immune-mediated pathophysiology. Subsequent evaluations ruled out infectious and neoplastic etiologies through extensive screening. Notable laboratory findings included transient elevation of serum creatinine and low complement levels (C3 0.199g/L, C4 0.025g/L). However, repeated screenings of the patient’s antinuclear antibody (ANA) profile and antiphospholipid antibodies demonstrated negative results.

Although the initial screening for SLE was negative, the newly onset of ketoacidosis in an adult, along with concurrent hypothyroidism, raises our suspicion that these two conditions might also be caused by an autoimmune mechanism. As expected, anti-glutamic acid decarboxylase antibody, insulin autoantibodies and thyrotropin receptor autoantibodies were all positive, leading to the diagnosis of autoimmune type 1A diabetes mellitus and autoimmune thyroiditis. Meanwhile, the observed thrombocytopenia was also suggestive of immune thrombocytopenia. Notably, significant reductions in complement levels were detected alongside a positive direct Coombs test. Additionally, renal impairment was identified, manifesting as hematuria, proteinuria, and elevated serum creatinine levels.

**Figure 3:**
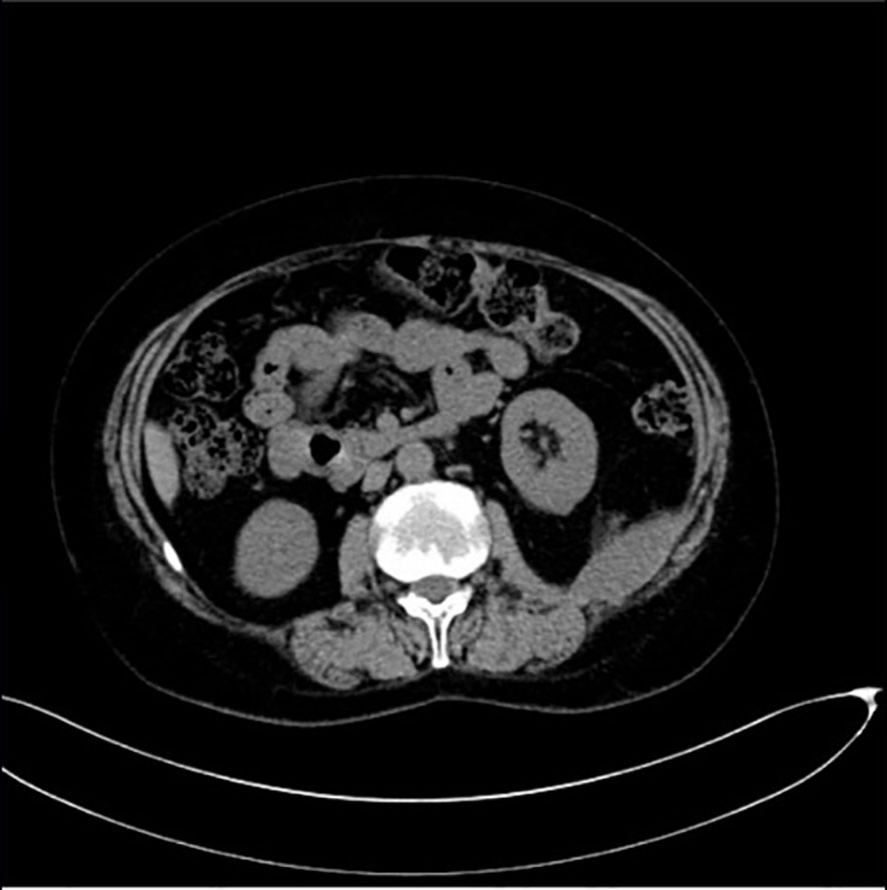
The follow-up CT demonstrates significant resolution of the hematoma.

Hence, the diagnosis of SLE was finally established and the patient received glucocorticoid therapy, resulting in a gradual improvement of her symptoms. Following discharge, the patient underwent an extended four-year follow-up period. Seroconversion to anti-dsDNA antibody positivity was detected six months post-discharge, providing conclusive immunological evidence for SLE. Due to the adverse drug reactions, the patient received multiple immunosuppressive agents, including hydroxychloroquine, sirolimus, azathioprine, and cyclophosphamide. Coagulation monitoring revealed persistent absence of FXIII inhibitors. Importantly, no hemorrhagic manifestations were observed throughout the therapeutic course, demonstrating effective hemostatic control through intensive immunosuppressive interventions (**Figure 3**).

## Discussion

As a rare coagulation disorder, FXIII deficiency demonstrates an estimated incidence of 1 in 2 million population (4). FXIII deficiency can be classified into congenital and acquired forms (5). Acquired FXIII (AH13) deficiency primarily affects elderly individuals and is characterized by severe hemorrhagic complications, including intramuscular and subcutaneous hemorrhages (6). Bleeding events are the leading cause of death in AH13 patient, leading to a mortality rate of approximately 20% (6), and lower FXIII activity is significantly associated with longer ICU stay and higher incidence of major bleeding (7).

The treatment of FXIII deficiency is often challenging. Whole blood, FFP, and cryoprecipitate can replenish deficient FXIII levels. However, replacement therapy alone is not successful in most cases (8). For patients of acquired FXIII deficiency, the primary objective is to eradicate autoantibodies. Plasmapheresis can remove autoimmune antibodies; but its therapeutic efficacy is transient (6). Thus, immunosuppressive agents, such as rituximab, are often administered to achieve sustained suppression of pathogenic autoantibodies. However, immunosuppressive therapy increases infection risks, potentially leading to fatal infectious complications (6).

Rituximab and bortezomib could suppress B cells, thereby inhibiting autoantibody production and ameliorating acquired FXIII deficiency. This regimen has been confirmed to be effective and safe in patients with acquired factor VIII deficiency (3). It has also been applied in AH13 patients at our center, where it showed promising efficacy. In this patient, hemoglobin levels gradually increased and FXIII activity returned to normal about one month after treatment, further supporting its efficacy of this treatment. However, although this treatment is effective in some patients, large-scale evidence is lacking due to the rarity of acquired FXIII deficiency, and further studies are still needed.

AH13 predominantly occurs secondary to autoimmune disorders (particularly systemic lupus erythematosus and rheumatoid arthritis), hematological malignancies, major surgical procedures, traumatic injuries and so on (9). Ichinose et al. summarize the clinical characteristics of 93 patients and found the two predominant reasons were idiopathic and autoimmune diseases (10). Notably, critical care modalities such as extracorporeal membrane oxygenation (ECMO) have been associated with progressive FXIII depletion (incidence rate 63%), potentially exacerbating coagulopathy in critically ill patients (11).

The patient’s symptom manifested at two weeks post-vaccination, raising the suspicion of vaccination-induced autoimmune dysfunction. Current evidence confirms that multiple vaccines could induce *de novo* autoimmune condition. The immune response may be modulated by both vaccine constituents and host-specific factors.

Specifically, COVID-19 vaccination has been linked immune thrombocytopenia, antiphospholipid syndrome, and even immune-mediated hepatitis (12–14). Interestingly, a population-based cohort study (n=9,258,803) further demonstrated a 1.16-fold increased risk of SLE following COVID-19 infection compared to controls (15).

However, there was no report supporting COVID-19 vaccine as an etiological factor for AH13. The multiple-systemic damage in this patient presents incongruent with vaccine-triggered autoimmunity paradigms, suggesting alternative etiologies. While vaccination may contribute to immune dysregulation in rare instances, acquired Factor XIII deficiency linked to recent vaccination remains low in the differential diagnosis hierarchy for this patient.

The multi-system damage, including endocrine, hematological, mucocutaneous and renal manifestations, coupled with the presence of multiple autoantibodies, prompted the consideration of SLE as a potential diagnosis. However, the negative ANA test may lead clinicians to hesitate in diagnosing SLE. Renal biopsy might provide histopathological confirmation of lupus nephritis, but the procedure was contraindicated due to the patient’s underlying hemorrhagic risks.

Although the 2019 EULAR classification criteria require a positive ANA titer for SLE classification (16), ANA-negative SLE remains a diagnostic challenge with an estimated prevalence of 2-9% (17). The 2012 Systemic Lupus International Collaborating Clinics (SLICC) classification criteria include anti-double-stranded DNA antibody, anti-beta 2-glycoprotein I, positive Coombs test, and low complement levels as serological markers, which was more suitable for ANA-negative SLE (18). In fact, this patient also meets the SLICC classification criteria and finally diagnosed with ANA-negative SLE.

Potential mechanisms for false-negative results may involve assay sensitivity limitations, alongside subthreshold antibody levels during early disease stages. Notably, previous studies found a significantly higher prevalence of ANA-negative SLE among patients previously treated with glucocorticoids (17). The pre-administration of glucocorticoids in this patient might also influence the detection accuracy.

The co-occurrence of SLE and acquired FXIII deficiency remains exceptionally rare, with only a few cases reported (19–24). In most cases, the diagnosis of SLE predating the occurrence of acquired FXIII deficiency. Only two cases have been reported in which patients with FXIII deficiency, presenting with severe bleeding as the initial manifestation, were ultimately diagnosed with SLE depending on the positive autoimmune antibodies (21, 22).

FXIII deficiency in ANA-negative SLE patients represents a novel clinical phenomenon without prior reports. This immunological paradox imposes additional pressure on clinicians to fully rule out alternative diagnoses.

Compared to ANA-positive SLE patients, seronegative cases might exhibit special clinical characteristics. For example, the ANA-negative SLE cohort exhibited an increased risk of thrombocytopenia compared to ANA-positive SLE (17). However, due to limited sample sizes in existing studies, further studies are need to delineate the precise phenotype and pathogenesis of ANA-negative SLE.

## Conclusions

In summary, this report firstly describes a patient initially manifesting severe hemorrhage of FXIII deficiency, who was ultimately diagnosed with the rare ANA-negative SLE following exhaustive evaluation. This case highlights the necessity for clinicians to deeply understand coagulation mechanisms and systematically screen for rare secondary etiologies.

## Data Availability

The original contributions presented in the study are included in the article/supplementary material. Further inquiries can be directed to the corresponding authors.
